# Partnership quality and maternal depressive symptoms in the transition to parenthood: a prospective cohort study

**DOI:** 10.1186/s12884-024-06757-9

**Published:** 2024-10-12

**Authors:** Cornelia E. Schwarze, Veronika Lerche, Stephanie Wallwiener, Sabina Pauen

**Affiliations:** 1 Department of Psychology, Developmental and Biological Psychology Unit, Heidelberg University, Hauptstraße 47-51, D-69117 Heidelberg, Germany; 2 Department of Psychological Methodology, Kiel University, Kiel, Germany; 3 Department of Obstetrics and Perinatal Medicine, Halle University, Halle, Germany

**Keywords:** Prenatal depression, Postpartum Depression, Maternal Mental Health, Partner Relationship Quality, Pregnancy, Cross-lagged panel analyses

## Abstract

**Background:**

Pregnancy and childbirth are critical life events which lead to significant changes in family structures and roles, thus having a substantial impact on partner relationship and maternal wellbeing. A dysfunctional partnership during this critical time of life has been associated with maternal depressiveness. However, sub-components of partnership quality and the causal relation with maternal symptoms of depression in the perinatal period have been sparsely studied so far. The current study aims to longitudinally assess the course of relationship quality and its sub-components from pregnancy to postpartum and to test a potential causal association with maternal symptoms of depression in the perinatal period.

**Methods:**

Differing from previous studies, partnership quality and symptoms of depression have been assessed prospectively and longitudinally from an early stage of pregnancy (second trimester) until six months postpartum. Cross-lagged panel models were applied to investigate a potential causal relationship between partnership quality and maternal depressive symptoms.

**Results:**

Relationship quality decreased significantly during the transition to parenthood (*p* < .05) with the steepest decline referring to tenderness (*p* < .001). We also found a substantial association of relationship quality and maternal depressiveness, but no indication for a clear causal direction of this association.

**Conclusions:**

Our results suggest that relationship quality and maternal depressiveness are substantially related in the perinatal period, thus pointing to the need of early prevention and intervention programs for peripartum women and their partners to prevent adverse outcome for the couple and the family.

**Supplementary Information:**

The online version contains supplementary material available at 10.1186/s12884-024-06757-9.

## Introduction

Of all mental health problems, depressive disorders are most common in the general population, ranging between 7% and 9% among females [1]. As suggested by multiple studies, women in the perinatal period are even more affected with 12% depressive disorders during pregnancy [1, 2] and 15% after childbirth (e.g. [3–5]). This indicates that the perinatal period is a critical time in life with an increased vulnerability for the onset, aggravation or reoccurrence of mental health problems (e.g. [6], but see [7], for some counter-evidence).

Maternal depression during pregnancy is a severe condition as it has been shown to affect fetal development, leading to lower birthweight, neonatal adaptation disorders and developmental delays [8, 9]. Recent meta-analyses confirm that maternal depression during pregnancy and beyond often has severe consequences for the psychological development of the child, showing associations with offspring socio-emotional development [10], externalizing and internalizing behavioral problems and poorer cognitive, language, motor, and adaptive behavior development during infancy, childhood, and adolescence [11].

On the other hand, maternal *post*partum depression is associated with impaired mother-child bonding [12, 13], an increased risk for insecure attachment [14], impaired mother-child interactions [15], developmental delays in the cognitive and language domain [16, 17], and increased levels of externalizing or internalizing behavioural problems [18]. Hence, it seems important to better understand what determines emotional stability in pregnant women and young mothers.

In this context, partner relationship quality may play an important role, as it has been shown to be negatively associated with depressive symptoms [19, 20]. Doss et al. [21] examined marital satisfaction, negative communication, relationship confidence, relationship dedication, conflict management and problem intensity in a sample of *N* = 132 recently married couples in their first eight years of marriage. The authors found deterioration in all of the six domains (except for relationship dedication) which suggests that partnership quality often decreases during the first years of relationship.

One factor contributing to these findings is childbirth. Pregnancy and childbirth are known as “critical life events” which lead to significant changes in family structures and roles, thus having a substantial impact on partner relationship satisfaction [22]. A meta-analysis by Mitnick et al. [23], including 37 studies, shows small to moderate decreases in relationship quality or satisfaction from pregnancy to the time after the child is born. A recent study also examined changes in relationship characteristics in different subgroups of mothers during and after pregnancy and confirmed the general finding of a significant decrease of relationship satisfaction following childbirth [24].

As pregnancy is a time of substantial bodily, hormonal, and social changes for women, they need to feel support with these challenges and those still lying ahead [25]. Hence, it has been suggested that poor partner relationship quality may provide a significant risk factor for the onset or reoccurrence of maternal mental health problems. Supporting this idea, Faisal-Cury et al. [26] recently reported that partner relationship quality predicted postpartum depression. But there is also evidence revealing that women with affective disorders prior to pregnancy carry an increased risk of developing an unfavourable peripartum partnership [27, 28]. Thus, effects in both directions, from depressive symptoms to less partnership satisfaction, and from partnership dissatisfaction to depressive symptoms seem plausible [29]. Dealing with a pregnant woman or a young mother suffering from depressive symptoms poses a challenge to the partner [30] who may respond with signs of withdrawal thus contributing to a decrease in partnership satisfaction. On the other hand, some women may be disappointed or dissatisfied with their partners following childbirth (e.g., due to a lack of support), eventually resulting in an increase of depressive symptoms. Hence, the causal direction of the observed relations between partner relationship quality and maternal depressive symptoms is not yet clear.

Taken together, we conclude that partner relationship undergoes considerable changes - especially in the transition to parenthood. Often, these changes point in a negative direction. However, previous studies that examined partnership satisfaction or –quality and perinatal maternal mood are usually based on correlational approaches, such as regression models [26, 27, 31, 32], which do not allow for causal conclusions in a strict sense. Studies combining a longitudinal design with the application of cross-lagged panel models that could actually inform us about the causal relation between partnership quality and perinatal maternal symptoms of depression are still rare. Notably, two studies assessing the association in a longitudinal design with cross-lagged analyses yielded contrasting findings: Thomas et al. (2019) found that relationship adjustment predicted subsequent levels of maternal depressive symptoms. In contrast, maternal depressive symptoms did not predict subsequent relationship adjustment. The study of Whisman et al. (2011) found that lower relationship adjustment was predictive of higher anxiety symptoms. Depressive symptoms were, however, not affected by prior relationship adjustment whereas they predicted subsequent relationship adjustment. However, both studies used very specialized samples, such as low-income ethnic US minorities [20] or women who already show a history of mental health problems [28]. Thus, these studies lack generalizability. Furthermore, both studies assessed partner relationship adjustment rather than partner relationship quality and its subcomponents such as conflict behavior, similarities, and tenderness.

The present work will help to fill this gap by using prospective longitudinal data of a Caucasian perinatal community sample and applying cross-lagged panel models to test the following hypotheses: (a) Partnership quality (and its subcomponents *tenderness*,* similarities*, and *conflict*) decrease from the pre- to the postnatal period. (b) Partnership quality and maternal symptoms of depression are associated not only during pregnancy but also following childbirth. (c) Partnership quality during pregnancy is related to maternal postpartum depression scores and vice versa. In addition, we use cross-lagged panel models to examine the directionality of the relation between partnership quality and maternal symptoms of depression. Thus, we investigate whether results reported in the literature which mainly refer to regression analyses differ from those obtained by cross-lagged panel models which provide a more direct way of testing causal hypotheses.

## Methods

### Study design and procedure

This prospective longitudinal study was conducted in the maternity departments of the University Hospitals Heidelberg and Tuebingen, Germany. We followed the requirements of the Helsinki declaration. Ethics approval was given by the ethics committee at Heidelberg University (project number S158/2016). Inclusion criteria for recruitment were maternal age > 18 years, a singleton pregnancy between the 20th and 27th gestational week (GW), and sufficient knowledge of the German language to read and answer our questionnaires. Exclusion criteria encompassed known fetal anomalies and malformations or multiple pregnancies. After participants were informed about the general goal of the research project and had given their written consent to participate in the study, the first digital baseline survey was completed. This baseline survey comprised the assessment of several demographic variables such as maternal age, employment status, and monthly household income. Furthermore, participants had to answer several questions relating to previous pregnancies and births.

In the further course, participants received online assessments eight times. Five times during pregnancy (Pre1 = 20th, Pre2 = 24th, Pre3 = 28th, Pre4 = 32nd and Pre5 = 36th GW) and three times following childbirth (Post1 = 7 days postpartum (pp), Post2 = 3 months pp, and Post3 = 6 months pp). Maternal symptoms of depression were assessed at every time point. Relationship quality was assessed twice, at 24th GW (Pre2) and 6 months postpartum (Post3). These two time points will in the following be termed T1 and T2. For our statistical analyses (cross-lagged panel design) we used data from T1 and T2, as these were the two measurement points when partner relationship quality and maternal depressive symptoms were both assessed at the same time. Participants who did not complete the relevant questionnaires (Partnership Questionnaire [PFB], Edinburgh Postnatal Depression Scale [EPDS]) at T1 or who completed less than 50% of the items of a questionnaire, were excluded from further analyses (for a detailed description of the questionnaires see chapter ‘Instruments’).

### Sample

Outpatient pregnant women from gestational week 20 on were recruited in the waiting area of the prenatal diagnostics division at the maternity departments of the University hospitals. 353 participants completed the relevant questionnaires (PFB and EPDS) at T1 (based on our criterion of at least 50% completed items for each questionnaire). The participants had an average age of *M* = 32.4 years (range = 21–45, *SD* = 4.7), which is matching the average age of childbearing women in Germany (31.8 years). The majority of the participants lived with their partners (98%). 73% of them were married, which is comparable with the German population (70%). Most women already had children (one child: 62%, two children: 14%, three or more children: 4%). Accordingly, the percentage of primiparous women was 20%. Most women were full-time employed (50%); 37% were employed part-time, and 12% of the women had no employment at the time of inclusion in our study. Compared to women in the German population, the participants in our sample were employed slightly more often (87% compared to 78%), whereas the percentage of those working only part time was lower (37% compared to 66%). The net household income was comparable to the average net household income in Germany (for more detailed information on the German population see data from the “Statistisches Bundesamt”, 2022).

A total of 113 participants (mean age = 33.0 years, range = 22–43, *SD* = 4.5; 23% primiparous women; 56% full-time and 32% part-time employed; 36% with a net household income of at maximum 2,000 €/month) completed both questionnaires at both measurement points (based on our criterion of at least 50% completed items for each questionnaire assessment). Overall, this subsample was largely comparable to the general German population. Among these participants, the mean percentage of completed items across participants amounted to a minimum of 99% for each questionnaire assessment and the vast majority of participants had complete data sets with no missing values (PFB at T1: 87%, EPDS at T1: 98%, PFB at T2: 91%, EPDS at T2: 96%).

### Instruments

#### Partnership Questionnaire (PFB)

The PFB [33] was applied to assess participants’ ratings of relationship quality and satisfaction with the partner. The questionnaire encompasses three subscales: (1) tenderness, (2) similarities and communication, and (3) conflict behavior. Each subscale contains ten items measured on a four-point scale (ranging from 0 to 3). While higher scores on the conflict behavior scale indicate less satisfaction, higher scores on the tenderness and similarities scales indicate a higher relationship quality. In a representative study conducted on a German population, good to very good reliabilities were achieved for all three subscales (Conflict: Cronbach’s $\:\alpha\:$ = 0.88; Tenderness: $\:\alpha\:$ = 0.91; Similarities and Communication: $\:\alpha\:$ = 0.85) and the overall scale ($\:\alpha\:$ = 0.93) [34]. Moreover, in confirmatory factor analyses based on a large German sample, Kliem et al. [35] found a good model fit for the factorial structure of the three subscales of the PFB. Kliem et al. [35] further showed that it is also justifiable to compute one overall total score. In our study, the internal consistencies of the different subscales and the overall scale (computed for the sample of 353 participants) were good at both measurement points: T1 (tenderness: $\:\alpha\:$ = 0.86, similarities: $\:\alpha\:$ = 0.82, conflict: $\:\alpha\:$ = 0.88, overall scale: $\:\alpha\:$ = 0.91) and T2 (tenderness: $\:\alpha\:$ = 0.90, similarities: $\:\alpha\:$ = 0.87, conflict: $\:\alpha\:$ = 0.89, overall scale: $\:\alpha\:$ = 0.94).

#### Edinburgh postnatal depression scale (EPDS)

The EPDS [36] is a widely used screening tool validated for assessing pre- and postpartum symptoms of depression [37, 38]. It has shown a good sensitivity and specificity of 0.96 and 1 [39]. The ten-item self-report questionnaire measures depressive symptoms during the past seven days. Every question is scored from 0 (no depressive symptoms) to 3 (severe depressive symptoms). For our analyses, we computed the sum across all items of the questionnaire after reversing the scores for inverted items (missing values were imputed by the mean across the remaining items of that participant). Higher values indicate a higher risk of minor or major depression. Internal consistency (computed for the sample of 353 participants) was good for both measurement points (T1: $\:\alpha\:$ = 0.85, T2: $\:\alpha\:$ = 0.87).

### Data analyses

For data preparation and analyses, we used the statistical program *R* [40]. For structural equation modeling (SEM), we applied the *R* package *lavaan* [41]. All significance tests were conducted as two-sided tests and based on a significance level of 5%.

First, we conducted attrition analyses to examine whether there were selective dropouts. We then calculated descriptive statistics for the two main variables of interest (relationship quality with its subscales, and maternal depressive symptoms), also reporting the percentage of participants meeting criteria for clinically significant depression.

Next, based on the sample of 113 participants who completed both questionnaires at both measurement points, we analyzed potential changes in relationship quality and maternal symptoms of depression from pregnancy (T1) to postpartum (T2), using repeated-measures *t*-tests. Then, we examined associations between relationship quality and maternal symptoms of depression at both measurement points using Pearson correlation coefficients.

In subsequent regression analyses with either relationship quality or maternal depression as criterion, we adjusted for several control variables: the dichotomized variables^1^ employment (full-time vs. part-time), income (low vs. high; 2.000 €/month served as cutoff score), as well as the metric variable maternal age. We also examined whether the control variables served as moderators of the relationship between relationship quality and maternal depression.

Finally, using cross-lagged panel models, we analyzed – based on the entire sample of 353 participants – whether relationship quality during pregnancy (T1) causally affects maternal postpartum symptoms of depression (T2) and/or vice versa. Cross-lagged panel models have the advantage of allowing a test of longitudinal associations between different measures which are independent of the stability and the concurrent associations of the measures (e.g. [42]). We compared a set of nested models. In these models, the parameters were progressively unconstrained. Model 1 was an autoregressive model, i.e., there were no lagged effects. The autoregressive path weights measure the stability of each variable across the two consecutive time points. In both Model 2 and Model 3 there was – in addition to the autoregressive paths – one cross-lagged effect. Model 2 included a cross-lagged effect from relationship quality at T1 to maternal depression at T2 and Model 3 the reverse cross-lagged effect from maternal depression at T1 to relationship quality at T2. Model 4, the reciprocal cross-lagged model, consisted of both cross-lagged effects. In all models, at both assessment points (T1, T2) the respective two variables were allowed to covary. With the scaled $\:\chi\:$^2^ difference tests we compared nested models [43]. As measures of relationship quality, we examined the overall scale and the three subscales of the PFB (tenderness, similarities, and conflict). Thus, in total, we analyzed four sets of cross-lagged panel models. For our cross-lagged panel analyses, we used SEM. More specifically, we used (standardized) manifest variables due to the restricted number of participants and large number of items in our study^2^. For parameter estimation, we relied on the estimator MLR which is a robust variant of a Maximum Likelihood estimator. Full information Maximum Likelihood was used to account for missing data.^3^,^4^

## Results

### Attrition analyses

Prior to the main analyses, we conducted attrition analyses. As mentioned earlier, 353 participants completed the relevant questionnaires (PFB and EPDS) at T1 (based on our criterion of at least 50% completed items for each questionnaire) and 113 participants completed both questionnaires at both measurement points (T1 and T2). We tested whether there were selective dropouts between T1 and T2, using independent sample *t*-tests and $\:\varvec{\chi\:}$^2^ tests to examine whether participants from whom data are available at both time points differed systematically from participants who only provided data at T1. There were no significant differences in the central variables, i.e., neither in relationship quality (tenderness, similarities, conflict) nor in maternal symptoms of depression (all *p*s > 0.507). Furthermore, there were no significant differences in the demographic variables either (parity, employment, income, maternal age; all *p*s > 0.094).

### Descriptive analyses and changes of partner relationship quality and maternal symptoms of depression from pregnancy to six months postpartum

Table 1 provides descriptive statistics for partnership relationship quality and maternal depressive symptoms during pregnancy (T1) and at six months postpartum (T2) for the sample of 113 women who completed both questionnaires at both measurement points. Among these women, 16% (*n* = 18) reached a level of depressive symptoms that was clinically relevant during pregnancy (T1), and 9% (*n* = 10) postpartum (T2), considering a cut-off score of 11 like recommended as result of a recent meta-analysis [44]. The results of the repeated-measures *t*-tests are reported in Table 2. Maternal symptoms of depression decreased slightly from pregnancy to six months after childbirth; however this difference failed to reach the level of significance (*t*[112] = 1.91, *p* = .059, *d*_*z*_ = 0.18).

**Table 1:** Means, standard deviations, and minimum and maximum values of all central variables

	M	SD	Min	Max
T1: PFB Overall	2.28	0.41	0.9	3.0
T2: PFB Overall	2.22	0.46	0.5	3.0
T1: Tenderness	2.18	0.50	0.9	3.0
T2: Tenderness	2.02	0.59	0.5	3.0
T1: Similarities	2.25	0.45	0.6	3.0
T2: Similarities	2.21	0.51	0.5	3.0
T1: Conflict	0.58	0.51	0.0	3.0
T2: Conflict	0.59	0.51	0.0	2.7
T1: Depression	5.50	4.90	0.0	19.0
T2: Depression	4.65	4.70	0.0	18.0

Note: The sample size (*N* = 113) is based on women who filled in at least 50% of the items of each of the two questionnaires (i.e., PFB and EPDS) at both time points

**Table 2:** Changes in partnership quality and symptoms of depression from the pre- to the postnatal period

Variable	M (SD)		t	*p*	95% CI		Cohen’s d_z_
	T1	T2			LL	UL	
PFB Overall	2.28 (0.41)	2.22 (0.46)	2.30	0.023	0.01	0.13	0.22
Tenderness	2.18 (0.50)	2.02 (0.59)	4.51	< 0.001	0.09	0.23	0.42
Similarities	2.25 (0.45)	2.21 (0.51)	1.12	0.265	-0.03	0.12	0.11
Conflict	0.58 (0.51)	0.59 (0.51)	−0.26	0.796	-0.08	0.06	−0.02
Depression	5.50 (4.90)	4.65 (4.70)	1.91	0.059	-0.03	1.75	0.18

Note: *CI* confidence interval, *LL* lower limit, *UL* upper limit. The sample size (*N* = 113) is based on women who filled in at least 50% of the items of each of the two questionnaires (i.e., PFB and EPDS) at both time points

Relationship quality decreased significantly from pregnancy to six months postpartum (*t*[112] = 2.30, *p* = .023, *d*_*z*_ = 0.22). In more detail, we found a significant decrease for the subscale *tenderness* (*t*[112] = 4.51, *p* < .001, *d*_*z*_ = 0.42), whereas the subscales *similarities* (*p* = .265) and *conflict* (*p* = .796) did not change significantly from pre- to postpartum.

### Association of partner relationship quality and maternal symptoms of depression

As reported in Table 3, we found significant negative associations of overall relationship quality and depressive symptoms both during pregnancy (T1: *r* = − .38, *p* < .001) and postpartum (T2: *r* = − .46, *p* < .001). Significant correlations with depressive symptoms were also found for all subscales of the PFB during pregnancy (T1;all *p*s < 0.05) and postpartum (T2;all *p*s < 0.001). Relationship quality and maternal symptoms of depression are also related longitudinally: We found a significant association of prenatal maternal depressiveness and relationship quality after childbirth (*r* = − .33, *p* < .001) as well as a significant association of relationship quality during pregnancy and maternal depressive symptoms postpartum (*r* = − .34, *p* < .001).

**Table 3:** Associations between relationship quality and maternal symptoms of depression

	1	2	3	4	5	6	7		8		9		10
1. T1: PFB Overall	−	0.83***	0.91***	−0.77***	−0.38***	0.74***	0.66***		0.66***		−0.60***		−0.34***
2. T1: Tenderness		−	0.74***	−0.37***	−0.24*	0.71***	0.78***		0.67***		−0.37***		−0.31***
3. T1: Similarities			−	−0.56***	−0.36***	0.62***	0.55***		0.65***		−0.40***		−0.32***
4. T1: Conflict				−	0.35***	−0.53***	−0.31***		−0.35***		0.72***		0.22*
5. T1: Depression					−	−0.33***	−0.29**		−0.27**		0.28**		0.50***
6. T2: PFB Overall						−	0.90***		0.91***		−0.76***		−0.46***
7. T2: Tenderness							−		0.83***		−0.46***		−0.41***
8. T2: Similarities									−		−0.52***		−0.43***
9. T2: Conflict											−		0.35***
10. T2: Depression													−

Note: The sample size (*N* = 113) is based on women who filled in at least 50% of the items of each of the two questionnaires (i.e., PFB and EPDS) at both time points. * *p* < .05. ** *p* < .01. *** *p* < .001

Furthermore, maternal symptoms of depression during pregnancy (T1) were highly correlated with maternal symptoms of depression postpartum (T2; *r* = .50, *p* < .001) and also the relationship quality overall scale (*r* = .74, *p* < .001) and the subscales of the questionnaire were highly correlated across all time points (all *p*s < 0.001).

In multiple regression analyses with either relationship quality or maternal symptoms of depression as criterion, we adjusted for the variables employment, income, and maternal age. Most importantly, relationship quality (overall score) still predicted postnatal maternal symptoms of depression (*p* = .006; see Table 4) and prenatal maternal symptoms of depression still predicted postnatal relationship quality (*p* = .003; see Table 5). In the Appendix, we also present separate analyses for the three subscales of the PFB (see Tables A1-A6 in the Appendix).

**Table 4:** Multiple regression results for postnatal depression (with predictor PFB overall)

Variable	b	95% CI for b		SE b	β
		LL	UL		
Intercept	11.51**	2.95	20.06	4.31	
PFB Overall	−4.44***	−6.80	−2.07	1.19	−0.38***
Age	0.09	−0.12	0.29	0.10	0.09
Fulltime Employment	0.50	−1.47	2.47	0.99	0.05
High Income	−0.17	−2.12	1.79	0.98	−0.02

Note. Fulltime Employment and High Income are dummy-coded variables with the value 1 indicating fulltime employment (0 = part-time) and high income (0 = low income). CI = confidence interval. LL = lower limit. UL = upper limit. The regression analyses are based on a sample without missings in the control variables (i.e., 98 participants). R^2^ = 0.15. $\:{R}_{adj}^{2}$ = 0.11. Adjusted R^2^ was computed according to Wherry Formula-1 [45] * *p* <.05. ** *p* < .01. *** *p* < .001

**Table 5:** Multiple regression results for PFB overall

Variable	b	95% CI for b		SE b	β
		LL	UL		
Intercept	2.56***	1.91	3.21	0.33	
Depression	−0.03**	−0.05	−0.01	0.01	−0.31**
Age	−0.01	−0.03	0.01	0.01	−0.12
Fulltime Employment	0.28**	0.10	0.46	0.09	0.30**
High Income	0.06	−0.13	0.24	0.09	0.06

Note. Fulltime Employment and High Income are dummy-coded variables with the value 1 indicating fulltime employment (0 = part-time) and high income (0 = low income). CI = confidence interval. LL = lower limit. UL = upper limit. The regression analyses are based on a sample without missings in the control variables (i.e., 98 participants). R^2^ = 0.20. $\:{R}_{adj}^{2}$ = 0.17. Adjusted R^2^ was computed according to Wherry Formula-1 [45]. * *p* <.05. ** *p* < .01. *** *p* < .001

Furthermore, the demographic variables did not serve as moderators of the effect of prenatal relationship quality on maternal postnatal depressiveness which applies to the overall scale as well as for the subscales (for all interaction effects *p*s > 0.202). The same was true for the effect of prenatal depressiveness on postnatal relationship quality (for all interaction effects *p*s > 0.159).

As mentioned in the introduction, regression analyses are frequently used for studying this issue, and often interpreted in causal terms even though cross-lagged panel models are needed to verify corresponding conclusions. Hence, we also conducted a corresponding analysis to clarify the meaning of the results presented so far.

### Cross-lagged panel models

**Fig. 1:**
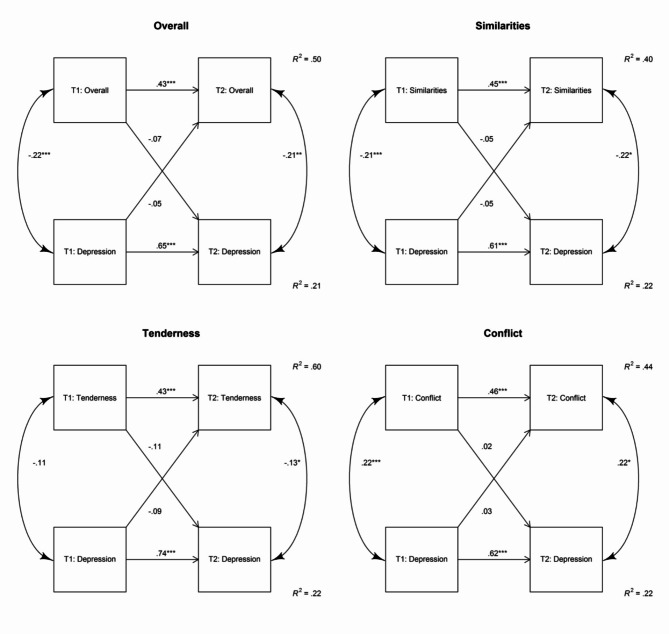
Reciprocal cross-lagged panel models (Model 4). * *p* < .05. ** *p* < .01. *** *p* < .001

Contrasting the findings from our regression models, the applied cross-lagged panel models did not reveal any significant effect between overall relationship quality and maternal symptoms of depression. We started with a comparison of Models 2 and 3 to Model 1, respectively. As illustrated in Table 6, for overall relationship quality neither Model 2 nor Model 3 revealed a significantly better fit than Model 1 (all *p*s > 0.220). Thus, the results from our study did not provide evidence supporting the assumption that partnership quality and maternal depressiveness are causally related. Table 7 shows the estimates for the best-fitting models (which is for each variable the autoregressive model). All the autoregressive effects were significant (all *p*s < 0.001) suggesting that there is a substantial stability of the measured constructs. The best predictor of maternal symptoms of depression at T2 were maternal depressive symptoms at T1, and the best predictor of relationship quality at T2 was relationship quality at T1. Furthermore, almost all synchronous associations were significant (exception: tenderness and depression at T1). For illustrative purposes, we additionally show the results of the reciprocal cross-lagged model (Model 4), see Fig. 1.

**Table 6:** Summary of model fit indices for competing cross-lagged models

PFB Scale	Model	χ^2^	df	*p*	TLI	CFI	RMSEA	AIC	BIC	∆χ^2^	*p*
Overall	1. Autoregressive	1.69	2	0.430	1.01	1.00	0.00	2,551.24	2,597.64		
	2. Cross-lagged (PFB on Depression)	0.66	1	0.415	1.02	1.00	0.00	2,551.96	2,602.23	0.97	0.320
	3. Cross-lagged (Depression on PFB)	0.80	1	0.372	1.01	1.00	0.00	2,552.36	2,602.62	0.91	0.340
	4. Reciprocal cross-lagged							2,553.35	2,607.48		
Tenderness	1. Autoregressive	3.64	2	0.162	0.96	0.99	0.05	2,561.73	2,608.12		
	2. Cross-lagged (PFB on Depression)	2.26	1	0.133	0.94	0.99	0.06	2,561.44	2,611.70	1.51	0.220
	3. Cross-lagged (Depression on PFB)	1.32	1	0.251	0.98	1.00	0.03	2,561.01	2,611.27	2.51	0.110
	4. Reciprocal cross-lagged							2,561.03	2,615.16		
Similarities	1. Autoregressive	0.92	2	0.632	1.05	1.00	0.00	2,579.95	2,626.35		
	2. Cross-lagged (PFB on Depression)	0.50	1	0.478	1.04	1.00	0.00	2,581.46	2,631.72	0.43	0.510
	3. Cross-lagged (Depression on PFB)	0.35	1	0.555	1.06	1.00	0.00	2,581.45	2,631.71	0.61	0.440
	4. Reciprocal cross-lagged							2,583.06	2,637.19		
Conflict	1. Autoregressive	0.32	2	0.851	1.06	1.00	0.00	2,570.96	2,617.36		
	2. Cross-lagged (PFB on Depression)	0.20	1	0.653	1.06	1.00	0.00	2,572.82	2,623.09	0.13	0.720
	3. Cross-lagged (Depression on PFB)	0.09	1	0.768	1.06	1.00	0.00	2,572.73	2,622.99	0.25	0.620
	4. Reciprocal cross-lagged							2,574.63	2,628.76		

Note: TLI = Tucker-Lewis index; CFI = comparative fit index; RMSEA = root mean square error of approximation; AIC = Akaike’s information criterion; BIC = Bayesian information criterion. The two rightmost columns show the results of the comparison of Models 2 and 3 with Model 1. Note that Model 4 is a fully saturated model without any degrees of freedom. Accordingly, for Model 4 the fit indices TLI, CFI, and RMSEA are not reported

**Table 7:** Parameter estimates of the best-fitting cross-lagged panel models (always Model 1)

Model	Parameter	Estimate	SE	*p*	95% CI	
					LL	UL
Overall	Loadings					
	Depression T1 on Depression T2	0.44	0.08	< 0.001	0.28	0.60
	Overall T1 on Overall T2	0.64	0.08	< 0.001	0.48	0.81
	Covariances					
	Depression T1 with Overall T1	−0.22	0.06	< 0.001	−0.34	−0.11
	Depression T2 with Overall T2	−0.21	0.08	< 0.01	−0.37	−0.05
Tenderness	Loadings					
	Depression T1 on Depression T2	0.43	0.09	< 0.001	0.27	0.60
	Tenderness T1 on Tenderness T2	0.75	0.05	< 0.001	0.64	0.85
	Covariances					
	Depression T1 with Tenderness T1	−0.11	0.06	0.050	−0.21	0.00
	Depression T2 with Tenderness T2	−0.13	0.06	0.040	−0.25	−0.01
Similarities	Loadings					
	Depression T1 on Depression T2	0.45	0.08	< 0.001	0.29	0.61
	Similarities T1 on Similarities T2	0.61	0.12	< 0.001	0.37	0.85
	Covariances					
	Depression T1 with Similarities T1	−0.21	0.06	< 0.001	−0.33	−0.10
	Depression T2 with Similarities T2	−0.22	0.09	0.010	−0.38	−0.05
Conflict	Loadings					
	Depression T1 on Depression T2	0.46	0.08	< 0.001	0.30	0.62
	Conflict T1 on Conflict T2	0.62	0.10	< 0.001	0.43	0.81
	Covariances					
	Depression T1 with Conflict T1	0.22	0.06	< 0.001	0.10	0.35
	Depression T2 with Conflict T2	0.22	0.10	0.020	0.03	0.41

Note: We report standardized path coefficients. *SE* standard error, *CI* confidence interval, *LL* lower limit, *UL* upper limit

## Discussion

The purpose of the present study was to assess the role of relationship quality and maternal symptoms of depression in the transition to parenthood in a perinatal Caucasian community sample to clarify a potential causal relationship between these two variables.

The main findings, resulting from our study are: (1) Partner relationship quality declines significantly from pregnancy to the postpartum phase. (2) Partner relationship quality and maternal symptoms of depression are correlated, both cross-sectionally and longitudinally. (3) Cross-lagged panel analyses do not indicate a causal relationship between both variables.

In the following, these findings will be discussed in detail.

### Decrease of partner relationship quality from pre- to postpartum

In accordance with previous studies [23, 24, 27], we found a significant decrease of partnership quality from pregnancy to postpartum, with the subscale *tenderness* showing a substantial decline. The size of this effect was small-to-medium. Asselmann et al. [27], who used the same relationship questionnaire, also reported a significant reduction of overall partnership quality after childbirth in women suffering from depression and anxiety prior to pregnancy but found the most significant change for the subscale *quarrelling*. Differences at the subscale level between studies may be associated with characteristics of the sample: While we tested a community sample of pregnant women, Asselmann et al. [27] tested women with a mental health diagnosis (depression and/or anxiety) prior to pregnancy. It is broadly known that subjects suffering from a manifest depressive disorder show increased interactional problems, especially in close relationships [46–48]. As a consequence, these women may experience more severe/frequent quarrelling with their partners in everyday life. Giving birth to a child and taking care of a newborn around-the-clock is a challenge for all primary caregivers, but it is plausible to assume that these stressors have an even stronger impact on mothers suffering from manifest mental health problems, such as depressive disorders, than for mothers in a mentally healthy condition.

The decrease in tenderness documented for the present sample can also be explained by the challenges of giving birth and taking care of a newborn infant: The primary caregiver, especially breastfeeding mothers, engages in intense and close physical contact with the baby, implicating that attention and tender care are usually focused on the offspring rather than the partner. A meta-analysis of Serati et al. [49] has shown that sexual activities decline during pregnancy and remain at comparably low levels 3–6 months following delivery. Apart from hormonal changes that contribute to this effect, it is well known that many women feel exhausted during pregnancy and beyond, and that some also suffer from bodily consequences of giving birth. Frequent sleep deprivation may further reduce the desire for close body contact with the partner. For many partners, this is not easy to cope with, thus potentially eliciting negative emotions [30]. In sum, there is often not enough time and energy left for sharing tenderness and sexuality among partners during the perinatal period, and especially during the early postnatal period - even in healthy couples.

Based on the reported findings, we conclude that the perinatal phase seems to be quite challenging for every couple. It often leads to a decrease in partnership satisfaction of women on multiple dimensions. Given that the observed decline in tenderness was only of small to medium size, it could be argued that most couples can tolerate it. It should be noted, however, that pregnant women who are vulnerable because they already suffer from depressive symptoms to some extent may experience this decline as further challenge which increases their risk for developing postpartum depression.

### Association of partner relationship quality and maternal symptoms of depression

Looking at the hypothesized association of partner relationship quality and maternal symptoms of depression, our data revealed that women who consider their relationship as poor show increased levels of depressive symptoms. Numerous studies report similar results, suggesting that poor partner relationship quality goes along with an increased severity of depressiveness during pregnancy and beyond [20, 26, 29, 50]. Findings obtained with a cross-lagged panel analysis did not suggest any causal relation between partnership quality and maternal depressive symptoms, however.

When interpreting these findings, it seems important to keep in mind that our sample was quite homogeneous with respect to partner relationship (98% living together with their partners; 73% of them married) and depressive symptoms (community sample, not including patients with known and diagnosed mental health problems). Nonetheless, we found a substantial association of both target variables at both measurement points T1 and T2, which replicates and extends existing work (e.g. [51, 26]).

At this point, we can only speculate about potential reasons for this association. One possible explanation refers to attachment theory [52]: According to Bowlby, children who are not securely attached to their caregivers develop a working model of social relations that is less based on trust than those who are securely attached [53]. As a consequence, they experience social stress more easily [54] and find it harder to feel supported by their partners [55] which increases the likelihood to develop depressive symptoms [56, 57]. Becoming a parent is known to bring back memories about one’s own childhood [58]. Hence, it seems plausible to assume that pregnant women with an insecure attachment style develop fears regarding their partnership more easily which increases the likelihood of experiencing a decrease in tenderness, as well as depressive symptoms during the perinatal period [59].

An alternative explanation for the observed association between tenderness and depressive symptoms refers to changes in hormonal balance of pregnant women: During pregnancy, women undergo substantial hormonal changes (see [60] for an overview). An imbalance in estradiol and progesterone has been shown to cause emotional instability and depressive symptoms in pregnant rodents [61] as well as in postpartum and premenstrual and postpartum mothers [62, 63]. At the same time, it is well-known that these hormones moderate women’s sexual drive [64], and that sexual activity is related to partnership satisfaction [65]. Hence, changes in estradiol and progesterone in the perinatal period might affect maternal mental well-being, and partnership quality at the same time. Another hormone, also of interest in this context is oxytocin: It is known to change from the pre- to the postnatal period (e.g. [66], see also [67] for a review). Furthermore, it seems to play a key role in social relations [68], thus linking depressive symptoms and partnership quality. Both explanations discussed so far may also jointly contribute to the robust association of maternal mood problems and partnership quality. Additional factors may elicit specific psychological dynamics in different couples, thus contributing to lack of evidence for a general causal relation in the cross-lagged panel analyses.

In line with findings from an earlier cross-lagged panel analysis (Thomas et al., 2019), we found concurrent and prospective correlations between measures of partner relationship quality (relationship adjustment) and maternal depressive symptoms. Differing from the previous study, we did not find any significant predictive effect of relationship quality on depressive symptoms, however. This may be due to multiple methodological differences between both studies: (1) Thomas and colleagues tested a sample of under-privileged families whereas the present sample was a well educated Caucasian sample with a high rate of (full time) employment. (2) The authors used different measures for the central variables than we did. (3) Significant prospective associations between partnership quality and maternal depressive symptoms were found only for measurement points when children were much older (i.e., from six years at T2 and seven years at T3).

Whisman et al. (2011) also examined relationship adjustment but in contrast to Thomas et al., they found that depressive symptoms affected subsequent relationship adjustment, but not vice versa. A key difference between Whisman et al.’s study and the one by Thomas et al. and our study is that they recruited a sample of women with a history of depression. More specifically, one inclusion criterion was the reporting of at least one lifetime episode of major depression. Thus, the distributions of the central variables (depressive symptoms and relationship adjustment) are likely different from our study sample, limiting comparability across studies. Interestingly, in line with the tendency observed in our study, Whisman et al. found that depressive symptoms assessed multiple times during pregnancy and postpartum showed a general decrease over time.

### Strengths and limitations

One strength of our study is the longitudinal study design with frequent measurements, including pregnant women from an early stage of pregnancy on (second trimester) and followed up until six months postpartum. Most other studies include pregnant women only in their last trimester of pregnancy (e.g. [69–71]).

Another strength is the prospective approach, including the assessment of symptom severity and relationship quality during pregnancy as well as in the postpartum period. Furthermore, the application of cross-lagged panel models – a study design and methodological approach that allows for causal conclusions – is still rare.

An important limitation of our study concerns the representativeness of our sample: The initial sample was recruited in two university towns with a mostly well-educated population. Consequently, it contains an increased percentage of highly educated women working full-time rather than part-time. Future studies may consider to extend recruitment to general hospitals outside university towns. Another factor limiting the representativeness of the present study is the large number of dropouts due to incomplete data sets. Even though this is quite common in online surveys [78], and even though the sample with complete data (*N* = 113) did not differ substantially from the sample with incomplete data (*N* = 353), nor from the German population in general, we cannot rule out that participants dropped out because they suffered from more severe mental health problems or because they experienced critical life events [72]. It is also possible that some women decided against participation or did not meet the inclusion criteria, because they were too stressed by preparing for childbirth or by adjusting to their life with a newborn, thus feeling a lack of time resources. Future studies could try to reduce dropout rates by including reminder calls, telephone interviews, or further expense allowances for study participation.

Even though we concede that these limitations clearly reduce the generalizability of the reported findings, we would also like to point out that factors limiting variance in important background variables lead to more conservative testing which suggests that the observed relation between partnership quality and depressive symptoms is likely to be quite robust. In addition, we want to indicate that the reported findings are well in accordance with previous findings in the literature. But only tests based on a sample that shows large variance in both target variables as well as control variables could clarify this point.

Future studies may also consider to assess hormonal changes and attachment style in addition, and to include methods that allow for a more fine-grained assessment of partnership experience and emotional states over time – e.g. by using ecological momentary assessment [73, 74], as this would help to better understand the mechanisms potentially underlying the observed relation between partnership quality and maternal depressive symptoms in the perinatal period.

### Implications

Consistent with previous research [20, 26, 32, 50, 51], our findings suggest that the transition to parenthood is associated with major changes in relationship quality which again documents the perinatal period as a time of an increased vulnerability.

To prevent mental health issues such as the development of manifest depressive disorders in pregnant women and young mothers, it is useful to offer targeted psychological prevention and intervention to help young families to successfully adjust to the new situation. Couple-focused interventions during the peripartum period have been shown to improve communication, mental health, and well-being in peripartum women [75–77].

## Electronic supplementary material

Below is the link to the electronic supplementary material.

Supplementary Material 1

## Data Availability

The datasets used and/or analyzed during the current study are available from the corresponding author on reasonable request.

## Footnotes

1 The variables were dichotomized so that the subgroups were sufficiently large to obtain an acceptable power.

2 We conducted additional analyses in which we also included the measurement models (assuming configural invariance over the two time points). The pattern of results was identical to the pattern of results for our analyses based on the manifest variables. Note, however, that given the suboptimal conditions (restricted sample size, large number of items), the fit indices were not satisfactorily and the results should only be interpreted with caution.

3 We conducted additional SEM analyses which were based on the restricted sample of 113 participants from who data from both time measurements were available. The main pattern of results was the same, but model fit was worse.

4 To assess the influence of missing data, we conducted additional analyses using a stricter 75% exclusion criterion (compared to the original 50%). This resulted in a minimal reduction in sample size (353 to 344 for cross-lagged models and 113 to 109 for the other analyses). Importantly, the results remained consistent across all analyses, with only a slight decrease in model fit for the SEM analyses.
