# Supplementary material for: Partnership quality and maternal depressive symptoms in the transition to parenthood: a prospective cohort study
Source: BMC Pregnancy Childbirth. 2024 Oct 12;24:664. doi: 10.1186/s12884-024-06757-9 (PMC11470716; doi:10.1186/s12884-024-06757-9)
Supplement: Supplementary file 1 — Supplementary Material 1 [file 12884_2024_6757_MOESM1_ESM.docx]

**APPENDIX**

**Article Title: Partnership Quality and Maternal Depressive Symptoms in the Transition to Parenthood:**

**A prospective Cohort Study**

**Authors: Cornelia E. Schwarze, Veronika Lerche, Stephanie Wallwiener, Sabina Pauen**

Table A1: Multiple Regression Results for Postnatal Symptoms of Depression with predictor Tenderness.

| Variable | b | 95% CI for b | | SE b | β |
| --- | --- | --- | --- | --- | --- |
|  |  | LL | UL |  |  |
| Intercept | 9.31* | 0.88 | 17.75 | 4.25 |  |
| Tenderness | −3.05** | −4.98 | −1.12 | 0.97 | −.32** |
| Age | 0.05 | −0.16 | 0.26 | 0.11 | .05 |
| Fulltime Employment | 0.10 | −1.86 | 2.07 | 0.99 | .01 |
| High Income | 0.06 | −1.93 | 2.06 | 1.00 | .01 |

**Note.** Fulltime Employment and High Income are dummy-coded variables with the value 1 indicating fulltime employment (0 = part-time) and high income (0 = low income). CI = confidence interval. LL = lower limit. UL = upper limit. The regression analyses are based on a sample without missings in the control variables (i.e., 98 participants). R^2^ = .11. $R_{adj}^{2}$ = .07. Adjusted R^2^ was computed according to Wherry Formula-1 [77]. * p < .05. ** p < .01.

Table A2: Multiple Regression Results for Postnatal Symptoms of Depression with predictor Similarities.

| Variable | b | 95% CI for b | | SE b | β |
| --- | --- | --- | --- | --- | --- |
|  |  | LL | UL |  |  |
| Intercept | 9.01* | 0.54 | 17.48 | 4.27 |  |
| Similarities | −3.30** | −5.49 | −1.11 | 1.10 | −.31** |
| Age | 0.08 | −0.12 | 0.29 | 0.11 | .08 |
| Fulltime Employment | 0.18 | −1.82 | 2.17 | 1.00 | .02 |
| High Income | 0.05 | −1.95 | 2.05 | 1.01 | .01 |

**Note.** Fulltime Employment and High Income are dummy-coded variables with the value 1 indicating fulltime employment (0 = part-time) and high income (0 = low income). CI = confidence interval. LL = lower limit. UL = upper limit. The regression analyses are based on a sample without missings in the control variables (i.e., 98 participants). R^2^ = .10. $R_{adj}^{2}$ = .07. Adjusted R^2^ was computed according to Wherry Formula-1 [77]. * p < .05. ** p < .01.

Table A3: Multiple Regression Results for Postnatal Symptoms of Depression with predictor Conflict.

| Variable | b | 95% CI for b | | SE b | β |
| --- | --- | --- | --- | --- | --- |
|  |  | LL | UL |  |  |
| Intercept | −1.35 | −8.55 | 5.85 | 3.63 |  |
| Conflict | 2.58** | 0.75 | 4.41 | 0.92 | .29** |
| Age | 0.14 | −0.07 | 0.35 | 0.11 | .14 |
| Fulltime Employment | −0.05 | −2.03 | 1.92 | 0.99 | −.01 |
| High Income | −0.51 | −2.55 | 1.52 | 1.03 | −.05 |

**Note.** Fulltime Employment and High Income are dummy-coded variables with the value 1 indicating fulltime employment (0 = part-time) and high income (0 = low income). CI = confidence interval. LL = lower limit. UL = upper limit. The regression analyses are based on a sample without missings in the control variables (i.e., 98 participants). R^2^ = .09. $R_{adj}^{2}$ = .06. Adjusted R^2^ was computed according to Wherry Formula-1 [77] . ** p < .01.

Table A4: Multiple Regression Results for Partnership Quality with Tenderness.

| Variable | b | 95% CI for b | | SE b | β |
| --- | --- | --- | --- | --- | --- |
|  |  | LL | UL |  |  |
| Intercept | 2.64*** | 1.75 | 3.53 | 0.45 |  |
| Depression | −0.03** | −0.06 | −0.01 | 0.01 | −.26** |
| Age | −0.02 | −0.05 | 0.01 | 0.01 | −.15 |
| Fulltime Employment | 0.21 | −0.03 | 0.45 | 0.12 | .17 |
| High Income | 0.08 | −0.17 | 0.34 | 0.13 | .07 |

**Note.** Fulltime Employment and High Income are dummy-coded variables with the value 1 indicating fulltime employment (0 = part-time) and high income (0 = low income). CI = confidence interval. LL = lower limit. UL = upper limit. The regression analyses are based on a sample without missings in the control variables (i.e., 98 participants). R^2^ = .12. $R_{adj}^{2}$ = .08. Adjusted R^2^ was computed according to Wherry Formula-1 [77]. ** p < .01. *** p < .001.

Table A5: Multiple Regression Results for Partnership Quality with Similarities.

| Variable | b | 95% CI for b | | SE b | β |
| --- | --- | --- | --- | --- | --- |
|  |  | LL | UL |  |  |
| Intercept | 2.50*** | 1.75 | 3.24 | 0.37 |  |
| Depression | −0.03* | −0.05 | −0.01 | 0.01 | −.24* |
| Age | −0.01 | −0.03 | 0.01 | 0.01 | −.12 |
| Fulltime Employment | 0.29** | 0.09 | 0.49 | 0.10 | .27** |
| High Income | 0.14 | −0.07 | 0.35 | 0.11 | .14 |

**Note.** Fulltime Employment and High Income are dummy-coded variables with the value 1 indicating fulltime employment (0 = part-time) and high income (0 = low income). CI = confidence interval. LL = lower limit. UL = upper limit. The regression analyses are based on a sample without missings in the control variables (i.e., 98 participants). R^2^ = .16. $R_{adj}^{2}$ = .13. Adjusted R^2^ was computed according to Wherry Formula-1 [77]. * p < .05. ** p < .01. *** p < .001.

Table A6: Multiple Regression Results for Partnership Quality with Conflict.

| Variable | b | 95% CI for b | | SE b | β |
| --- | --- | --- | --- | --- | --- |
|  |  | LL | UL |  |  |
| Intercept | 0.45 | −0.29 | 1.20 | 0.37 |  |
| Depression | 0.03** | 0.01 | 0.05 | 0.01 | .28** |
| Age | 0.00 | −0.02 | 0.03 | 0.01 | .04 |
| Fulltime Employment | −0.34** | −0.54 | −0.13 | 0.10 | −.32** |
| High Income | 0.06 | −0.16 | 0.27 | 0.11 | .05 |

**Note.** Fulltime Employment and High Income are dummy-coded variables with the value 1 indicating fulltime employment (0 = part-time) and high income (0 = low income). CI = confidence interval. LL = lower limit. UL = upper limit. The regression analyses are based on a sample without missings in the control variables (i.e., 98 participants). R^2^ = .18. $R_{adj}^{2}$ = .15. Adjusted R^2^ was computed according to Wherry Formula-1 [77] . ** p < .01.
